# The prognostic significance of semi-quantitative metabolic parameters and tumoral metabolic activity based on ^123^I-MIBG SPECT/CT in pretreatment neuroblastoma patients

**DOI:** 10.1186/s40644-025-00858-0

**Published:** 2025-03-31

**Authors:** Ziang Zhou, Xu Yang, Guanyun Wang, Xiaoya Wang, Jun Liu, Yanfeng Xu, Kan Ying, Wei Wang, Jigang Yang

**Affiliations:** Department of Nuclear Medicine, Beijing Friendship Hospital, Capital Medical University, 95 Yong An Road, Xi Cheng District, Beijing, 100050 China

**Keywords:** Neuroblastoma, Semi-quantitative, Prognosis, ^123^I-MIBG, Asphericity

## Abstract

**Purpose:**

To assess the prognosis predictive value of semi-quantitative metabolic parameters and tumoral metabolic activity based on ^123^I-meta-iodobenzylguanidine (MIBG) SPECT/CT in pretreatment neuroblastoma (NB) patients.

**Methods:**

A total of 50 children (25 girls, 25 boys, median age 37 months, range 1-102 months) with newly diagnosed NB, consecutively examined with pretherapeutic ^123^I-MIBG SPECT/CT between 2018 and 2024, were included in this retrospective study. The semi-quantitative metabolic parameters and activity of primary tumor were measured, including Tmax/Lmax, Tmean/Lmean, Tmax/Lmean, Tmax/Mmax, Tmean/Mmean and asphericity (ASP). The ratio was maximum or mean count of primary tumor, liver and muscle. Clinical data and image-related factors was recorded as well. The outcome endpoint was event-free survival (EFS). Independent predictors were identified through univariate and multivariate logistic regression analyses. Receiver operating characteristic (ROC) and Kaplan Meier analysis with log-rank test for EFS were performed.

**Results:**

Median follow-up was 42 months (range 2.5–74 months; 4 patients showed disease progression/relapse, 7 patients died). The univariate and multivariate Cox regression analysis demonstrated that bone/bone marrow metastasis [95% confidence interval (CI): 1.051, 18.570, *p* = 0.043], Tmax/Lmax (95% CI: 1.074, 1.459, *p* = 0.004) and ASP (95% CI: 2.618, 273.477, *p* = 0.006) were independent predictors of EFS. The Kaplan Meier survival analyses demonstrated that Tmax/Lmax undefined$\:>$]]>6 and ASP $\:>$undefined]]>34% and with bone/bone marrow metastasis had worse outcomes.

**Conclusion:**

In this exploratory study, pretherapeutic ^123^I-MIBG image-derived semi-quantitative metabolic parameters and tumor asphericity provided prognostic value for EFS in NB patients. Tmax/Lmax $\:>$undefined]]>6 and ASP $\:>$undefined]]>34%, along with the presence of bone/bone marrow metastasis, could be considered as supplementary factors alongside existing ones.

## Introduction

Neuroblastoma (NB), the most frequent malignancy solid tumor in children, preferentially originates from adrenal medulla or para-spinal ganglia [1]. NB shows a remarkable heterogeneity [2]. In infants, spontaneous resolution of NB can occur even in cases of extensive disseminated disease, whereas older patients with metastatic or biologically aggressive localized disease have a long-term survival rate of less than 50% despite receiving intensive multimodal therapy [3].

Thus, risk stratification, an approach used for more than two decades, is critical to guide therapy. Event-free survival (EFS) and overall survival (OS) outcomes are influenced by key prognostic risk factors, including age at diagnosis, International Neuroblastoma Risk Group (INRG) stage, MYCN status, histopathology, and ploidy status [4]. In the past few years, there has been a rise in the identification of novel biomarkers such as sequential circulating tumor DNA or tumor specific mRNA transcripts [5]. These promising biomarkers have the potential to enhance and optimize future risk classification systems for NB. However, the image related prognostic or risk factors are few.

The NCCN NB panel recommends ^123^I-meta-iodobenzylguanidine (MIBG) SPECT/CT imaging should be used to assess for metastatic disease due to the its high specificity and high sensitivity [6]. The modified Curie score and the International Society of Pediatric Oncology Europe Neuroblastoma (SIOPEN) score are the two most widely used semiquantitative scoring systems for interpreting ^123^I-MIBG imaging [7–10]. However, at present, there is no quantitative parameter obtained from ^123^I-MIBG that can independently forecast a patient’s prognosis regardless of the tumor stage. Among other parameters, low ratio of mean count of tumor/muscle based on ^123^I-MIBG SPECT/CT was correlated with early relapse of NB, T/Mmean = mean count of tumor/mean count of muscle [11]. Moreover, the tumor’s aggressiveness can be assessed by quantifying the asphericity (ASP) of its metabolic activity distribution, which can serve as a surrogate indicator for the biological nature of the tumor [12]. Recently, the concept of ASP has been successfully transferred to SPECT imaging and quantifying the heterogeneity and semi-quantitative tumor metabolic activity, which appears promising for the prediction of therapy outcome in patients with metastatic gastroenteropancreatic neuroendocrine neoplasms [13].

Hence, the objective of this study was to assess the predictive importance of semi-quantitative indexes obtained from pretherapeutic ^123^I-MIBG SPECT/CT, along with clinical information and image-related factors in relation to event-free survival (EFS). EFS duration was measured from the date of 123I-MIBG SPECT/CT imaging until the first documented progression event or death from any cause.

## Materials and methods

### Patients

**Fig. 1:**
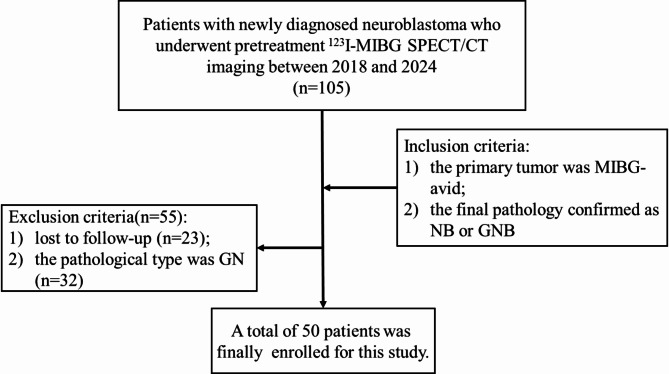
Flow chart for the inclusion and exclusion criteria of patients with NB

This retrospective study included 50 consecutively examined children, including 25 girls and 25 boys; with a median age of 37 months (range 1–102 months). These children were newly diagnosed with NB and referred to Beijing Friendship Hospital of Capital Medical University for pretherapeutic ^123^I-MIBG SPECT/CT between in 2018 and 2024. The eligibility criteria were as follows: (1) ^123^I-MIBG SPECT/CT were performed in all patients; (2) without treatment (including surgery or polychemotherapy) before the examination; (3) the primary tumor was MIBG-avid and the final pathology confirmed as NB or ganglioneuroblastoma (GNB). Patients were excluded when they were lost to follow-up and the pathological type was ganglioneuroma (GN) (Fig. 1). The general information and clinical data of patients was recorded. Laboratory parameters were also obtained at the time of diagnosis. Normal maximum values for serum neuron-specific enolase (NSE) ranged from 0 to 25 ug/L and the serum lactate dehydrogenase (LDH) ranged from 110 ~ 295U/L. MYCN amplification status as well as chromosomal aberration 1p36, 11q23 were assessed by fluorescence in situ hybridization. We could also record the treatment and date of death or disease progression or the last day of follow-up through patient’s medical charts or telephone calls. The primary outcome was event-free survival (EFS), which encompassed progression-free survival and overall survival.

The study was conducted in accordance with the 2013 revision of the Declaration of Helsinki. It received approval from the Institutional Board of Beijing Friendship Hospital of Capital Medical University (No. 2022-P2-321-03), and individual consent for this retrospective analysis was waived.

### ^123^I-MIBG SPECT/CT acquisition

All patients, include newborn, received thyroid blockade by oral administration of Lugol’s solution to protecting the thyroid from unnecessary irradiation [14]. The ^123^I-MIBG dosage was determined following the guidelines of the Pediatric Committee of the European Association of Nuclear Medicine (EANM) [15]. SPECT imaging was performed 24 h after tracer injection in addition to both anterior and posterior planar whole-body projections. The tomography was promptly conducted subsequent to the acquisition of the planar images [16]. The criteria for selecting the tomographic visual field were as follows: If the planar images were negative, the tomographic site was selected for surgery. If positive, it was chosen for suspicious lesions. The SPECT/CT was Siemens Symbia T16 (Siemens Healthcare, Germany) with a low-energy all-purpose collimator. The whole-body planar scintigraphic images were obtained at a rate of 5 cm/min in a matrix of 256 × 256. The SPECT followed a circular orbit spanning 360°, employing a matrix size of 128 × 128, zoom 1.6. Frames were acquired at intervals of every 6 degrees over a duration of 20 s. CT scans were acquired for attenuation correction, localization, and resolution restoration with the following parameters: 80 kV and 130 mAs.

### ^123^I-MIBG SPECT/CT semi-quantification

The images were evaluated by two experienced nuclear medicine physicians with more than 15 years. The regions of interest (ROIs) for primary tumors were manually delineated using 3D Slicer software (version 4.13.0, Boston, Massachusetts, United states) [17]. Subsequently, the maximum count rate (Tmax), average count rate (Tmean), ASP of the primary tumor were calculated. The ASP of the primary tumor was defined as

$$\:\text{A}\text{S}\text{P}=∛H-1,\:\text{w}\text{i}\text{t}\text{h}\:\text{H}=\frac{1}{36\pi\:}\:\frac{{S}^{3}}{{V}^{2}}$$

undefined

Where S and V are the surface area and volume of the MTV, respectively [18].

The maximum and average liver count rate, Lmax and Lmean respectively were defined based on a 30-ml sphere place in physiological liver tissue. Similarly, the maximum and average muscle count rates were determined by placing a 10-ml sphere in the physiological uptake site on both sides of the lumbar 2 vertebral body [11]. The final count for Mmax and Mmean was obtained by averaging the counts from both sides. The tumor to liver count rate ratio (TLCRR: Tmax/Lmax, Tmax/Lmean, Tmean/Lmean) and tumor to muscle count rate ratio (TMCRR: Tmax/Mmax and Tmean/Mmean) were calculated [19].

### Statistical analysis

Statistical analysis was performed using SPSS 27.0 (IBM Corporation, Armonk, NY, USA) and R 4.3.3 (R Foundation for Statistical Computing, Vienna, Austria). Continuous variables were described using medians and interquartile ranges (IQR), categorical various were described using frequency and percentage. The risk of adverse outcome in matched cohort was assessed using the univariate Cox regression analysis with hazard ratio (HR) and its 95% confidence interval (CI) calculation. All variables with p$\:\le\:$0.05 in the univariate Cox regression were included in the multivariate Cox regression suing the forward stepwise method to identify independent prognostic predictors. Kaplan-Meier curves with log-rank scores were used in the survival analysis. The Jordan index was used to determine the optimal cutoff values, chosen through the receiver operating characteristic (ROC) analysis of area under the curve (AUC) parameter and its 95% CI. The sensitivity, specificity, positive predictive value (PPV), and negative predictive value (NPV) were also computed. The statistical tests were conducted using a two-tailed approach, and a p-value of less than 0.05 was considered indicative of statistical significance.

## Results

The clinical characteristics of all patients are summarized in Table 1. Events occurred in 11 of the 50 patients (22%), including relapse in 2, progression in 2, death without relapse/progression in 2 and 5 died during follow-up. The median follow-up duration of the survivors was 42 months (range 2.5 to 74 months; IQR: 438 to 2098 days). The majority of the primary tumors (78%) were located in the abdominal region, with 39 of 50. In addition, 7 of 50 (14%) were found in the mediastinum and 4 of 50 (8%) in the pelvic cavity. The IDRFs was positive in 60% of the patients and the occurrence of bone/bone marrow metastasis was observed in 33 of 50 (66%) patients. 23 of 50 (46%) patients received neoadjuvant chemotherapy before undergoing surgery.

**Table 1:** Patients characteristics

Parameters	Value (%)
**N**	50
**Gender**	
Male	25 (50%)
Female	25 (50%)
**Age at diagnosis**	
Median (months)	37
$\:<$18 months	18 (36%)
$\:\ge\:18\:\text{m}\text{o}\text{n}\text{t}\text{h}\text{s},\:<5\:\text{y}\text{e}\text{a}\text{r}\text{s}$	18 (36%)
$\:\ge\:$5 years	14 (28%)
**Primary tumor site**	
Abdomen	39 (78%)
Mediastinum	7 (14%)
Pelvic	4 (8%)
**IDRF**	
Positive	30 (60%)
Negative	20 (40%)
**IDRF number**	1.8 (0, 3), range: 0–7
**Curie score**	3.3 (0, 2), range: 0–28
Extent of MIBG uptake	
More than muscle and less than liver	19 (38%)
More than liver	31 (62%)
**Bone/bone marrow metastases**	
No	33 (66%)
Yes	17 (34%)
**Histology**	
NB	33 (66%)
GNB	17 (34%)
**NSE (ng/ml)**	89.1 (23.65, 58.6), range: 18–830
Number	37 (74%)
No information	13 (26%)
**LDH (U/L)**	361.57 (228, 345), range: 191–1870
Number	30 (60%)
No information	20 (40%)
**MYCN**	
Normal	33 (66%)
Amplified or gained	4 (8%)
No information	13 (26%)
**1p36 aberration**	
No	30 (60%)
Yes	2 (4%)
No information	18 (36%)
**11q23 aberration**	
No	30 (60%)
yes	5 (10%)
No information	15 (30%)
**Asp**	0.3 (0.18, 0.33), range − 0.5–1.5
**Treatment**	
Neoadjuvant chemotherapy	23 (46%)
Surgery	50 (100%)
Post-operative chemotherapy	31 (62%)
Radiotherapy	13 (26%)

IDRF: image-defined risk factors; MIBG: meta-iodobenzylguanidine; NB: neuroblastoma; GNB: ganglioneuroblastoma; NSE: neuron-specific enolase; LDH: lactate dehydrogenase; ASP: asphericity

### Prognostic relevance of parameters

In the univariate Cox regression analysis, showed in Table 2, number of IDRFs (95% CI: 1.102, 1.886, *p* = 0.008), Curie score (95% CI: 1.029, 1.165, *p* = 0.004), bone/bone marrow metastasis (95% CI: 1.852, 26.696, *p* = 0.004), Tmax/Lmax (95% CI: 1.124, 1.558, *p* = 0.001), Tmax/Lmean (95% CI: 1.075, 1.320, *p* = 0.001) and ASP (95% CI: 5.529, 261.054, p$\:<$0.001) were significant predictors of EFS. Age at diagnosis (95% CI: 0.834, 17.949, *p*=0.084) showed a tendency to significance.

**Table 2:** Univariate and multivariate logistic regression analysis with respect to event-free survival

Characteristics	Univariate analysis		Multivariable analysis	
	HR (95% CI)	*p*	HR (95% CI)	*p*
Age at diagnosis	3.870 (0.834, 17.949)	0.084		
Number of IDRFs	1.442 (1.102, 1.886)	**0.008**		
Extent of MIBG uptake	2.337 (0.505, 10.825)	0.278		
Curie score	1.095 (1.029, 1.165)	**0.004**		
Bone/bone marrow metastasis	7.031 (1.852, 26.696)	**0.004**	4.419 (1.051, 18.570)	**0.043**
Histology	4.511 (0.564, 36.087)	0.156		
maximum diameter of the tumor	1.076 (0.924, 1.253)	0.347		
Tmax/Lmax	1.323 (1.124, 1.558)	**0.001**	1.252 (1.074, 1.459)	**0.004**
Tmean/Lmean	1.228 (0.806, 1.871)	0.338		
Tmax/Lmean	1.191 (1.075, 1.320)	**0.001**		
Tmax/Mmax	1.048 (0.977, 1.123)	0.189		
Tmean/Mmean	0.954 (0.789, 1.140)	0.602		
ASP	37.992 (5.529, 261.054)	**0.000**	26.755 (2.618, 273.477)	**0.006**

Values in bold type are statistically significant (p$\:<$0.05)

HR: hazard ratio; CI: confidence interval; IDRF: image-defined risk factors; MIBG: meta-iodobenzylguanidine;

The multivariate Cox regression analysis demonstrated that bone/bone marrow metastasis (95% CI: 1.051, 18.570, *p* = 0.043), Tmax/Lmax (95% CI: 1.074, 1.459, *p* = 0.004) and ASP (95% CI: 2.618, 273.477, *p* = 0.006) were independent predictors of EFS (Table 2).

**Fig. 2:**
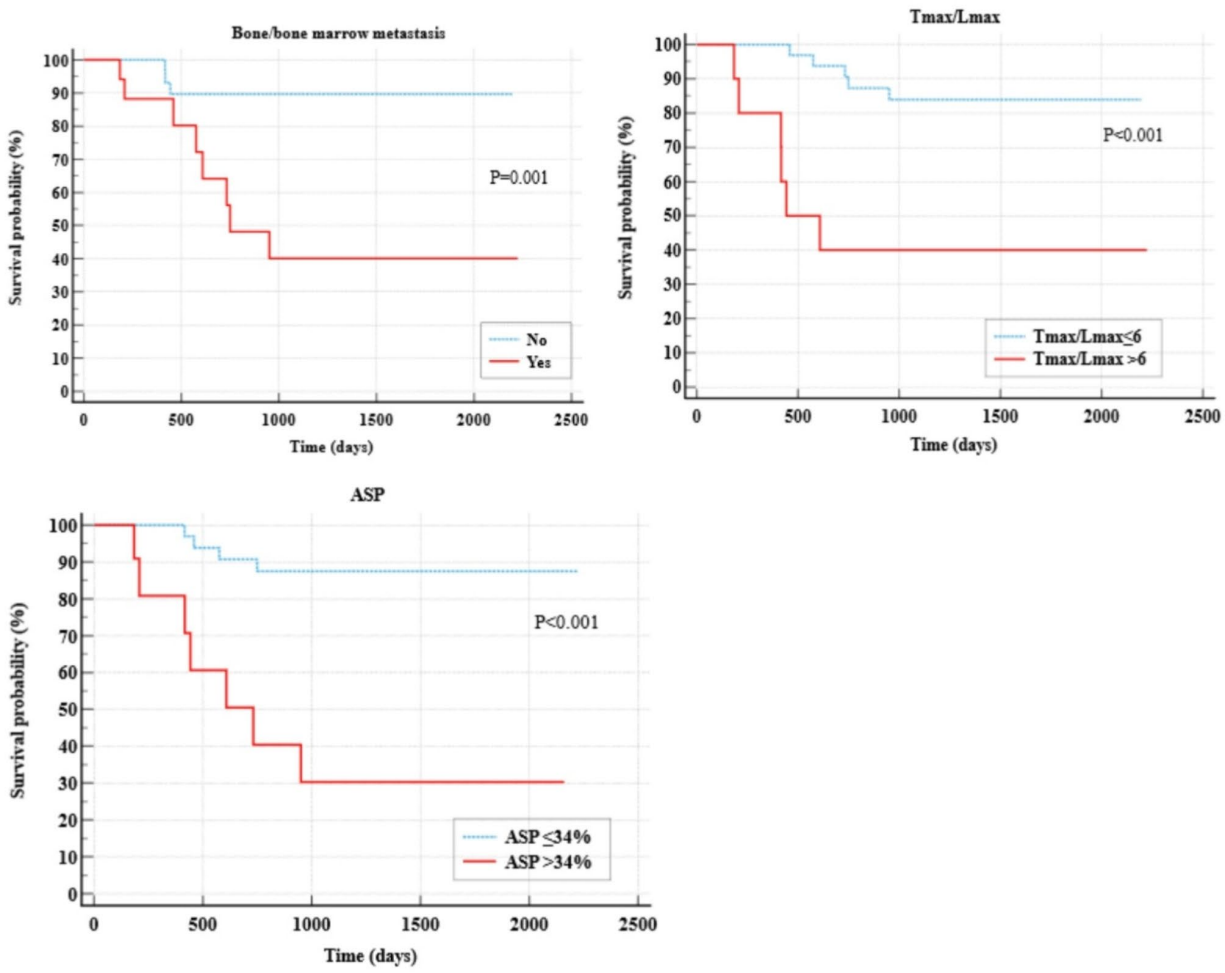
Kaplan Meier survival curves with log-rank test for event-free survival for selected parameters with p$\:\le\:$ 0.05 in the univariate Cox regression

ROC analysis for metric parameters identified Tmax/Lmax $\:>$undefined>]]6 and ASP $\:>$undefined]]>34% as optimal cut-off values. The sensitivity, specificity, PPV and NPV of Tmax/Lmax were 54.5%, 89.7%, 60% and 87.5% respectively (Table 3). The diagnostic accuracy measures for ASP showed a sensitivity of 63.6%, specificity of 89.7%, PPV of 63.6%, and NPV of 89.7%. Based on the Kaplan-Meier analysis, patients with high ASP (> 34%) had a mean EFS of 1011 days (95% CI: 522.718, 1499.019), while those with low ASP had a mean EFS of 2015 days (95% CI: 1822.205, 2207.062). The log-rank test showed a significant difference between the two groups (*p* < 0.001). Patients with high Tmax/Lmax (> 6) had a mean EFS of 1117 days (95% CI: 522.757, 1681.643), while those with low Tmax/Lmax had a mean EFS of 1954 days (95% CI: 1759.914, 2148.729). The log-rank test showed a significant difference between the two groups (*p* < 0.001). Patients with bone/bone marrow metastasis had a mean EFS of 1241 days (95% CI: 797.440, 1686.218), while those without bone/bone marrow metastasis had a mean EFS of 2012 days (95% CI: 1815.771, 2208.091). The log-rank test showed a significant difference between the two groups (*p* = 0.001). The Kaplan-Meier curves are shown in Fig. 2.

**Table 3:** The optimal cut-off value of significant parameters

	Cut-off	Sensitivity (%)	Specificity (%)	PPV (%)	NPV (%)
Tmax/Lmax	6.278	54.5	89.7	60.0	87.5
ASP	0.342	63.6	89.7	63.6	89.7

## Discussion

**Fig. 3:**
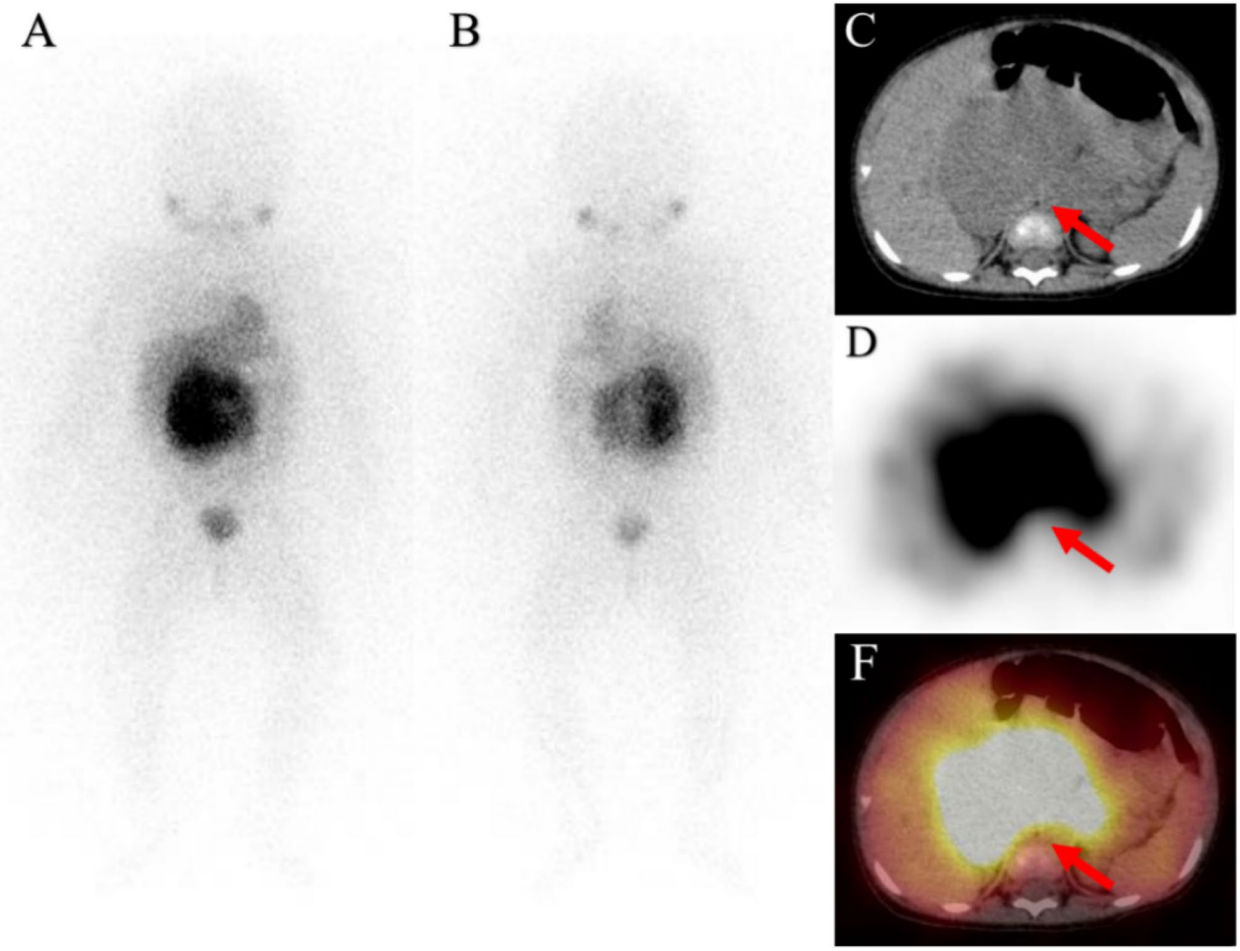
A ten-month old girl was diagnosed as having abdomen neuroblastoma, which showed intense activity on the initial staging 123I-MIBG whole-body and axial images (**A**, anterior, **B**, posterior, **C**, CT, **D**, SPECT, **E**, fusion). The number of IDRFs was 5, the maximum diameter of tumor was 10.2, Curie score was 1, no bone/bone marrow metastasis was observed. Tmax/Lmax ratio was 3 and ASP was 30%. The patient is still alive with a follow-up of 1880 days

**Fig. 4:**
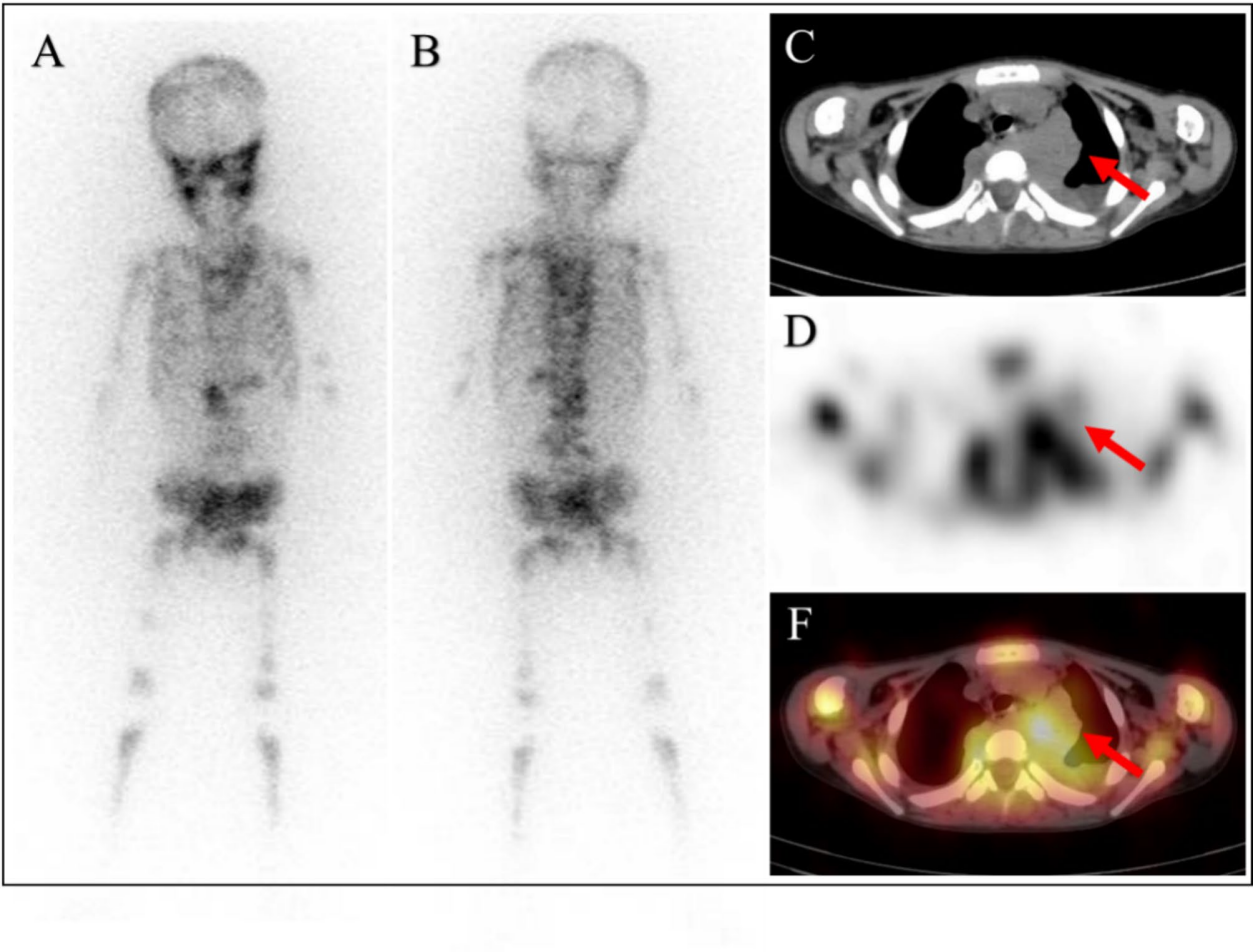
A four years old boy was diagnosed as having left-chest paraspinal neuroblastoma. The 123I-MIBG whole-body and axial SPECT/CT images revealed intense tumor activity (**A**, anterior, **B**, posterior, **C**, CT, **D**, SPECT, **E**, fusion). The IDRFs counts were 7, with a maximum tumor diameter of 5.1. The Curie score measured 28, and there were indications of metastasis in the whole-body bone/bone marrow. A ratio of 10 was observed in Tmax/Lmax, while ASP reached 150%. Unfortunately, the patient died after 184 days

This retrospective study evaluated the potential of ^123^I-MIBG SPECT/CT semi-quantitative metabolic parameters in pretreated NB patients. Our research revealed that with bone/bone marrow metastasis, Tmax/Lmax $\:>$undefined]]>6 and ASP $\:>$undefined]]>34% are independent predictors of EFS (Figs. 3 and 4).

To the best of our knowledge, metastases are present at diagnosis in around 50% of patients, with bone and bone marrow being the major sits of involvement [20, 21]. The occurrence of bone/bone marrow metastases and recurrence in NB poses a significant obstacle in clinical treatment, requiring a comprehensive comprehension and focused therapeutic interventions. The HR 4.419 for bone/bone marrow metastases represents a four-fold increased risk of progression or relapse in the pretreatment NB patients. Most patients already presenting with metastatic disease at the time of diagnosis, suggesting that metastatic occurs early during disease progression, which is consistent with our findings. In our research, 3 out of 33 (9%) patients without bone/bone marrow died compared to 8 of 17 (47.1%) with bone/bone marrow involvement. In the study conducted by Gunes Gundem et al., malignant clones emerge early during embryogenesis, rapidly diversify, and disseminate across local and distant metastatic sites prior to disease diagnosis, thereby unraveling the intricate networks of disease dissemination during relapse in response to therapy [22].

Variable approaches have been used, from descriptive, qualitative image analysis to semiquantitative of MIBG scans, that allow estimation of individual tumor loads. The Curie or SIOPEN scores, which is based on planar imaging and widely utilized in various centers, represents the most commonly employed semi-quantitative method [23–25]. However, it fails to accurately reflect tumor metabolism. Previous studies have demonstrated that the T/Mmean ratio of the delay-relapse group was found to be significantly higher than that of the early-relapse group based on SPECT-derived parameters. Fendler et al. demonstrated that in pediatric patient with NB, strong ^123^I-MIBG uptake indicates unfavorable histopathology. The semiquantitative tumor to liver count rate was used as a tool [19]. However, there has been no specific assessment conducted to determine its predictive significance in NB. Our research findings also indicate that a Tmax/Lmax ratio more than 6 is associated with an unfavorable prognosis. The hazard ratio of 1.252 per unit signifies a tenfold increase in the risk of EFS among patients with the highest measured Tmax/Mmax (13.2) compared to those with the lowest Tmax/Mmax (0.9). The findings of our study also provide evidence that a higher degree of tumor uptake, indicated by an increased Tmax value, is associated with a more favorable prognosis. However, the SUVmax did not predict EFS in Yadgarov’s research [26]. This may be attributed to the limited sample size of their patient population.

ASP of the primary tumor was another significant predictor of EFS in the univariate and multivariate regression analysis. A high ASP is considered an indicator of a more aggressive tumor biology, as it occurs due to either low tracer avidity within the tumor or a heterogeneous outer surface with spiculated MTV. Ivayla and Julian et al. found that the novel parameter of pretherapeutic FDG uptake asphericity seems to provide better predictive value for PFS and OS in non-small cell lung cancer when compared to SUV, MTV, total lesion glycolysis, and solidity [27, 28]. Nonetheless, these studies relied on PET imaging scans. Wetz et al. were the first to utilize ASP in SEPCT imaging, and their findings indicated that an elevated ASP observed during somatostatin receptor imaging is a strong predictor of non-response to peptide receptor radionuclide therapy among patients diagnosed with metastatic gastroenteropancreatic neuroendocrine neoplasms [13]. In the same year, Rogasch et al. demonstrated the effectiveness of ASP in analyzing ^123^I-MIBG SPECT/CT images for NB patients. Their findings revealed that pretherapeutic ASP analysis of tumoral metabolic activity could accurately predict the likelihood of progression or relapse in children undergoing current therapy [29]. An additional preliminary investigation, reported by Yadgarov, revealed that imaging parameters associated with the metabolic activity and roundness of tumors demonstrated predictive significance for event-free survival among high-risk NB patients. The findings of their study indicate that asphericity ≥ 65% can serve as an additional prognostic factor in conjunction with the existing ones [26]. The ASP value of our study was 34%, which was more consistent with the results of Rogasch et al., who reported a value of 32%. It could be because Yadgarov’s study was focused on high-risk patients. The limitation of the two studies was their small patient cohorts.

In our study, the number of IDRFs, Curie score and Tmax/Lmean were had significant in univariate Cox regression. Previous studies had showed that IDRFs ≥ 4 was significant indicators of poor prognosis and should be considered in protocol planning, instead of IDRF presence or absence [30, 31]. Several previous studies found no significant prognostic impact of the initial MIBG score or did not address outcome [32, 33]. However, Boris et al. reported that the Curie and the SIOPEN scores were highly correlated and equally reliable and predictive [25]. Our research demonstrated that Curie score was a significant prognosis factor. Recently, therapies such as anti-GD2 antibody treatment and ^131^I-MIBG radionuclide therapy have been introduced. In our study nearly half of the patients underwent neoadjuvant chemotherapy before surgery. All patients who underwent both chemotherapy and radiotherapy survived, indicating that a combination of treatment methods might be essential to enhance survival rates and cure rates. Kitamura et al. were reported that the early-relapse group and the delay-relapse group exhibited a noticeable disparity in Tmax/Lmean, which was similar to our study [11]. Tmax/Lmean could be associated with poorer survival outcomes in NB patients.

There are some limitations in our study. Firs of all, it is a retrospective single-center study. Thus, a large multicenter prospective study should be conducted to confirm the present study results. Secondly, our study included patients with varying INSS stages who received diverse treatment regimens, deviating from the standardized protocol. This deviation may have influenced the outcomes. Another limitation is that despite the large number of patients, few children had events. The possible reasons are as follows: some patients may not be well followed up because of death, Unlike CT or MRI, ^123^I-MIBG scans cannot be readily assessed at any given time and in some cases, patients with severe conditions may have undergone surgery or chemotherapy prior to the initial MIBG examination. This may have introduced selection bias.

## Conclusion

In order to improve the outcome of patients with NB, more sophisticated and efficient prognosis predictors are required. In this exploratory study, imaging parameters related to tumoral metabolic activity, such as ASP and Tmax/Lmean derived from pretherapeutic ^123^I-MIBG SPECT/CT, as well as the metastatic pattern, presence or absence of bone/bone marrow metastasis, were identified as independent prognostic factors for EFS. Those factors may provide assistance for NB in individualized risk stratification and therapy management.

## Data Availability

No datasets were generated or analysed during the current study.

## Abbreviations

NB: Neuroblastoma
MIBG: Meta-iodobenzylguanidine
ASP: Asphericity
EFS: Event-free survival
OS: Overall survival
INRG: International Neuroblastoma Risk Group
SIOPEN: Society of Pediatric Oncology Europe Neuroblastoma
GNB: Ganglioneuroblastoma
GN: Ganglioneuroma
NSE: Serum neuron-specific enolase
LDH: Lactate dehydrogenase
EANM: European Association of Nuclear Medicine
IQR: Interquartile ranges
CI: Confidence interval
HR: Hazard ratio
ROC: Receiver operating characteristic
AUC: Area under the curve
PPV: Positive predictive value
NPV: Negative predictive value
